# El desafío de la sostenibilidad de los programas ampliados de inmunizaciones

**DOI:** 10.26633/RPSP.2017.160

**Published:** 2017-12-20

**Authors:** Ernesto Báscolo, Camilo Cid, Juan Pablo Pagano, María Soledad Urrutia, Amalia Del Riego

**Affiliations:** 1 Organización Panamericana de la Salud Organización Panamericana de la Salud Washington, DC Estados Unidos de América Organización Panamericana de la Salud, Washington, DC, Estados Unidos de América.

**Keywords:** Integración de sistemas, acceso universal a servicios de salud, sistemas de salud., Systems integration, universal access to health care services, health systems., Integração de sistemas, acesso universal a serviços de saúde, sistemas de saúde.

## Abstract

**Objetivo.:**

*Analizar la sostenibilidad y la integración de los programas prioritarios en el marco de la Estrategia para el acceso universal a la salud y la cobertura universal de salud (Salud Universal) de la Organización Panamericana de la Salud de 2014*.

**Métodos.:**

*Se llevó a cabo una revisión no sistemática de la bibliografía reciente enfocada al análisis de la integración y la sostenibilidad*.

**Resultados.:**

*El principal resultado que se extrae de la bibliografía revisada es la necesidad de abordar la sostenibilidad de tal modo que se superen los límites de cualquier análisis restringido a la sostenibilidad financiera. Aunque la integración y la sostenibilidad no se interpretan de forma homogénea, sobresale un abordaje que contempla la integración como un factor facilitador del proceso de sostenibilidad del PAI*.

**Conclusiones.:**

*La efectividad de las estrategias de integración del PAI depende en buena medida de que se consideren la presencia, el fortalecimiento y la transformación de las estructuras organizativas e institucionales que faciliten la participación de los actores con capacidad técnica y política para garantizar sus procesos de implementación. De esta forma, se podrán ponderar los procesos políticos que legitiman una agenda de inclusión del PAI en la Estrategia de salud universal y, por tanto, como parte integrante del fortalecimiento de los sistemas de salud*.

## Abstract (es)

**Objective.:**

Analyze the sustainability and integration of priority programs in the framework of the Pan American Health Organization’s 2014 Strategy for Universal Access to Health and Universal Health Coverage (Universal Health).

**Methods.:**

A non-systematic review was carried out of the recent literature focused on analysis of integration and sustainability.

**Results.:**

The main result extracted from the literature review is the need to address sustainability in a way that goes beyond the limits of any analysis restricted to financial sustainability. Although integration and sustainability are not interpreted uniformly, an approach that includes integration as a facilitating factor of the EPI sustainability process does stand out.

**Conclusions.:**

Effectiveness of EPI integration strategies depends to a large extent on considering the presence, strengthening, and transformation of organizational and institutional structures that facilitate the participation of actors with the technical and political ability to ensure its implementation processes. Thus, political processes can be weighed that legitimize an agenda to include EPI in the Universal Health strategy and, accordingly, as an integral part of health systems strengthening.

## RESUMO

**Objetivo.:**

Avaliar a sustentabilidade e a integração do Programa ampliado de imunização (PAI) como parte da estratégia de acesso universal à saúde e cobertura universal de saúde da Organização Pan-Americana da Saúde (OPAS) de 2014.

**Métodos.:**

Uma revisão não sistemática da literatura científica recente foi realizada dando enfoque à análise da integração e sustentabilidade do PAI.

**Resultados.:**

O principal achado é a necessidade de contemplar a sustentabilidade além da análise de sustentabilidade financeira. Embora a integração e a sustentabilidade não sejam interpretadas de forma homogênea na literatura, são destacadas as virtudes de uma abordagem que conceba a integração como um fator facilitador do processo de sustentabilidade do PAI.

**Conclusões.:**

A efetividade das estratégias de integração do PAI depende em grande parte da existência, fortalecimento e transformação das estruturas organizacionais e institucionais facilitando a participação dos interessados com capacidade técnica e política para garantir os processos de implementação. Desse modo, será possível examinar com atenção os processos políticos que legitimam a agenda de inclusão do PAI na estratégia de saúde universal e, portanto, como parte integrante do fortalecimento dos sistemas de saúde.

Con el establecimiento del Programa Ampliado de Inmunización (PAI) de las Américas en 1977 comenzó una de las intervenciones de salud pública prioritarias más exitosas de la Región (1). Entre los mayores éxitos atribuibles al PAI se encuentran la certificación de la erradicación de la poliomielitis de la Región en 1994 (2), la eliminación de la rubéola, del síndrome de la rubéola congénita y del sarampión en 2015 y 2016, respectivamente, y la disminución de la mortalidad infantil por enfermedades prevenibles con vacunas en la mayoría de los países (3).

El desarrollo y la implementación del PAI han tenido distintas modalidades. Mientras que en algunos países se han vinculado tempranamente con el desarrollo del primer nivel de atención y el fortalecimiento de los sistemas de salud, en otros el PAI es un programa totalmente vertical con dificultades para su sostenibilidad, porque no se ha incorporado a un proceso de transformación y sostenibilidad del sistema de salud en su conjunto (4, 5).

Algunas características del PAI lo colocan en una situación ventajosa para su sostenibilidad en comparación con otros programas prioritarios: la vacunación como bien público es la actividad en salud que goza de mayor aceptación social y su fuerte sustento técnico basado en la evidencia le brinda un gran impulso y legitimidad en los países. Sin embargo, el PAI no es ajeno a la necesidad de disponer de recursos estables, suficientes y sostenibles para su financiamiento — que, además, comparten otros programas prioritarios — como elementos determinantes de su sostenibilidad.

El contexto epidemiológico y sociodemográfico en el cual se insertan los programas de salud influye en su sostenibilidad y difiere entre países y regiones. La Región de las Américas se caracteriza por una avanzada transición demográfica, con una esperanza de vida promedio de 77 años y una tasa de fecundidad global (2,0 hijos por mujer) muy cercana al umbral de la de reemplazo (6). El envejecimiento de la población y la mayor prevalencia de enfermedades no transmisibles generan presiones financieras crecientes sobre los sistemas de salud y acentúan la preocupación por la sostenibilidad de los programas, especialmente en países de menores ingresos y con mayores tasas de fecundidad (7).

En este sentido, la dependencia de la ayuda externa y la presencia de cooperantes han sido muy dispares en la Región. En algunos países, la participación de actores como la GAVI (Alianza Global para las Vacunas) ha sido determinante y continúa siéndolo, aunque en los países con fuerte presencia de la cooperación internacional se espera que ésta disminuya progresivamente en los próximos años. Los países que se van “graduando” entran en un período de transición en el financiamiento de sus programas prioritarios que requiere comprometer más recursos locales. Esta retirada programada de la ayuda internacional ha despertado el interés por la sostenibilidad de los programas de inmunizaciones, así como por la de otros programas prioritarios a escala internacional, lo cual sitúa a este tema en un lugar prominente de la agenda (3).

Sin embargo, el interés por la sostenibilidad de los programas de salud no surge con la caída de la ayuda internacional. Este debate se remonta a la década de los ochenta y a la preocupación de que ciertas intervenciones no logren perdurar mucho tiempo (8, 9). Además, ya desde la década de los noventa, el análisis de la sostenibilidad trasciende la dimensión financiera e incorpora otros factores de orden organizativo, institucional y político (9, 10).

Entendiendo que los desafíos de la sostenibilidad van más allá de la necesidad de sustituir fuentes de financiamiento externas por fuentes locales (3, 11), se ha adoptado una perspectiva más amplia y multidimensional (12-14), que incluye el análisis de las bondades de la integración de los programas prioritarios en los sistemas de salud a través de un diseño “horizontal” que supera las virtudes de las estructuras tradicionales de los diseños “verticales” (14-16).

En la Región es necesario abordar la sostenibilidad de estos programas de tal forma que se pueda articular un enfoque integral de los problemas que plantea en el marco de las características institucionales y de las políticas de transformación de los sistemas de salud de los países.

La Estrategia para el acceso universal a la salud y la cobertura universal de salud (Salud Universal) de la OPS de 2014 representa una referencia indispensable para considerar los retos más importantes de los sistemas de salud. En esta Estrategia la segmentación y la fragmentación de los programas de salud se identifican como dos desafíos centrales, que “se perpetúan por la falta de capacidad regulatoria dentro de los sistemas de salud, al igual que por la verticalidad de algunos programas de salud pública y la falta de integración de los mismos a nivel de la prestación de los servicios” (17). En este contexto, la sostenibilidad y la integración de los programas prioritarios, y en particular del PAI, son temas de especial relevancia.

Este trabajo tiene como objetivo analizar la sostenibilidad y la integración de los programas prioritarios en el marco de dicha Estrategia.

## MATERIALES Y MÉTODOS

Con este objetivo se llevó a cabo una revisión no sistemática de la bibliografía en que recientemente se han analizado las dimensiones de la integración y la sostenibilidad. La búsqueda bibliográfica se realizó en Google académico y en Pub-Med utilizando los siguientes criterios de selección: a) artículos publicados a partir del año 2000, período en que se combina una novedosa producción teórica desde la teoría neoinstitucional, utilizada en los artículos seleccionados, junto con un debate global sobre los desafíos de la sostenibilidad de programas prioritarios desde agencias internacionales (11, 18); b) los que incorporan la sostenibilidad o la integración como temas centrales; c) los que incluyen una robusta revisión bibliográfica, ya sea teórica o como aplicación a estudios de casos, y d) los que contienen un marco analítico como producto explícito.

Los artículos seleccionados se presentan en este estudio considerando sus objetivos, su abordaje metodológico y las dimensiones estudiadas. De forma complementaria, se analizó su contenido considerando los siguientes tres ejes analíticos: 1) en qué medida y cómo son abordadas la sostenibilidad o la integración como parte del objeto de análisis, 2) cómo se consideran en ellos los factores políticos e institucionales como facilitadores de la sostenibilidad, y 3) la forma como se caracterizan los procesos de cambio. Finalmente, estos ejes se utilizaron para interpretar la sostenibilidad y la integración del PAI en el marco de la Estrategia para el acceso universal a la salud y la cobertura universal de salud.

## RESULTADOS

En el cuadro 1 se presentan los artículos seleccionados y se identifican sus objetivos, su abordaje metodológico y las dimensiones analizadas en ellos. Los objetivos coinciden en la necesidad de disponer de un marco analítico que supere un análisis restringido a la sostenibilidad financiera. Sobre los métodos, sólo dos de los estudios incluidos en esta revisión basan su producción en revisiones bibliográficas sistemáticas (14, 15). En cuanto a las dimensiones de los análisis, un rasgo común es que estas dimensiones están asociadas con dos tipos de visiones: una más relacionada con las funciones de un sistema de salud (12, 14, 15) y otra con mayor apego a los procesos (como adopción, difusión y asimilación) (13, 19). Por último, la relación entre sostenibilidad e integración está presente en todos los artículos y emerge de forma clara en aquellos que incorporan explícitamente en el análisis de los problemas y las características de los sistemas de salud (12, 15). A continuación, se describe la forma como se han abordado los tres ejes analíticos en los estudios incluidos en esta revisión.

### La sostenibilidad y la integración como objeto de análisis

Los estudios de Oberth y Whiteside (12) y de Pluye y colaboradores (13) consideran la sostenibilidad como un resultado buscado y entendiéndola como la dimensión principal, y la integración, como una condición necesaria o como potencialmente sinónima de la sostenibilidad. Ambos estudios se diferencian tanto por las propuestas como por el abordaje utilizado en el análisis de la relación entre integración y sostenibilidad. El primero propone diferentes dimensiones de sostenibilidad para reconocer distintos aspectos que deberían tenerse en cuenta al intentar garantizar la sostenibilidad de los programas de vih y el segundo construye un marco analítico que sintetiza la bibliografía publicada sobre este tema y propone diferentes fases de sostenibilidad progresivas.

**CUADRO 1.** Marcos de análisis seleccionados

Autores, año (referencia)	Objetivo principal	Abordaje metodológico	Dimensiones del análisis	Relación sostenibilidad-integración
Oberth, Whiteside, 2016 (11)	Conceptualizar las dimensiones de la sostenibilidad en un contexto de caída de la ayuda internacional con un enfoque en casos de transición financiera de programas de VIH/SIDA	Basado en un debate bibliográfico que combina bibliografía teórica y experiencias nacionales y donantes	Plantean cinco dimensiones de sostenibilidad: Financiera Epidemiológica Politica Estructural Programática Derechos humanos	No se aborda directamente esta relación, aunque en la dimensión programática se deja planteada la interrogante: ¿tiene sentido el programa específico en un sistema integrado basado en la atención primaria de salud?
Shigayeva, Coker, 2014 (14)	Analizar y conceptualizar la sostenibilidad y la relación entre las nociones de sostenibilidad e integración	Revisión sistemática de la bibliografía sobre marcos conceptuales o analíticos de sostenibilidad y estudios empíricos	Plantean seis características de un programa sostenible: Capacidad para gobernar, liderar y gestionar Recursos y capacidad de planificar e implementar Adaptación, renovación, flexibilidad Construir relaciones e interacciones Mostrar resultados y alcanzar objetivos	La integración se plantea como un facilitador de la sostenibilidad, aunque no es el único. Sin embargo, el papel de la integración con otros componentes del sistema de salud es específico del contexto y difícil de predecir
Atun, et al., 2009 (18)	Construir un marco analítico de integración en torno a las funciones criticas del sistema de salud	Revisión de estudios teóricos y empíricos sobre adopción y difusión de innovaciones en sistemas de salud	Plantean la integración como un proceso evolutivo en tres etapas: Adopción Difusión Asimilación	Necesariamente la adopción, difusión y asimilación requieren alinear la intervención con las funciones del sistema de salud. Se supone que esta integración definirá la sostenibilidad del programa
Gruen, et al., 2008 (13)	Construir un marco empírico para entender la sostenibilidad y aplicarlo cuando ésta se planifica	Revisión sistemática de la bibliografía sobre sostenibilidad de programas de salud	Interacción entre el problema de salud, los “drivers” (incluyendo actores como los donantes) del programa y el programa mismo. A las 3 interacciones las denomina: Ciclo de calidad Economía política Definición del problema	No aborda directamente la integración como un factor de sostenibilidad. Sí se menciona que la sostenibilidad es más probable cuando los componentes del sistema están conectados y alineados
Pluye, et al., 2004 (12)	Analizar bajo qué estructuras organizativas se genera la sostenibilidad y en qué etapa comienza	Revisión de trabajos empíricos sobre sostenibilidad de programas	Plantean dos dimensiones del análisis de sostenibilidad: Estructuras sociales que sostienen a los programas: Organizacionales (rutinas) Institucionales (estándares) Temporalidad: La sostenibilidad no se da en una etapa final, es un proceso concomitante a la implementación de un programa	En la conceptualización de rutinas y estándares se mezclan elementos de integración de los programas. La presencia de rutinas estandarizadas es una forma de integración de una intervención

Los trabajos de Gruen y colaboradores (14) y de Shigayeva y Coker (15) también consideran la sostenibilidad como principal preocupación, pero en ellos se percibe con más claridad que la integración es un factor facilitador de la primera. Sin embargo, la integración adquiere un significado diferente en cada caso. Gruen y colaboradores entienden que es preciso explorar las interacciones entre los impulsores de cambio y los componentes del programa en cada contexto particular para analizar los determinantes de la sostenibilidad (14). Las estructuras formales y las relaciones son importantes, pero también lo son las relaciones informales que guían el comportamiento humano. En cambio, Shigayeva y Coker entienden la integración como parte de las características de los programas y tienen en cuenta la interacción entre los componentes de los programas en el seno de la organización en la cual se insertan y con el resto de las organizaciones del sistema de salud (15).

Por último, el estudio de Atun y colaboradores se circunscribe al análisis exclusivo de la integración, que en él se define como el proceso de extensión, patrón, tasa de adaptación y eventual asimilación de una intervención en cada una de las funciones críticas del sistema de salud, un concepto similar al de sostenibilidad empleado en los otros estudios (19).

### Factores políticos e institucionales como motores de cambio

Las categorías políticas e institucionales están presentes en todos los artículos revisados, aunque con diferencias importantes entre ellos. En el de Oberth y Whiteside se incluyen la sostenibilidad política (el apoyo de actores críticos) y la sostenibilidad programática (la integración de los programas en el sistema de salud) como ingredientes necesarios para el abordaje integral de la sostenibilidad, aunque no se trata la relación entre tales dimensiones políticas e institucionales (12).

Por su parte, el de Pluye y colaboradores se centra en el análisis institucional al asumir la sostenibilidad como un proceso de institucionalidad creciente, que se interpreta como producción de rutinas y estandarización de nuevos programas o intervenciones. El proceso político, con menor visibilidad, emerge en la medida en que se considera que la participación y la relación entre actores sociales son algunos de los factores que influyen en el proceso de institucionalización. Sin embargo, dado el carácter teórico del trabajo, no se visibilizan dimensiones institucionales del sistema de salud como parte de las dimensiones analíticas (13).

En el estudio de Gruen y colaboradores se hace hincapié en el proceso político para explicar la influencia de actores, sus intereses, valores, concepciones y la capacidad de movilizar recursos, elementos todos ellos que se conciben como impulsores de cambio sobre la sostenibilidad de los programas (14).

Finalmente, en los estudios de Shigayeva y Coker (15) y de Atun y colaboradores (19) se explicitan tanto los procesos políticos —entendidos como participación de actores y liderazgo en los procesos de cambio—, como las dimensiones institucionales, y se analizan programas y sistemas de salud. En el de Shigayeva y Coker el abordaje institucional hace mención especial de la gobernanza, de los servicios de salud y de los modelos de financiamiento como aspectos centrales de la institucionalidad sectorial (15).

### Caracterización del proceso de cambio

Aunque en todos los estudios revisados se supone que la sostenibilidad y la integración se han de interpretar de forma dinámica, se aprecian diferentes formas de analizarlas. Mientras que Oberth y Whiteside sólo identifican diferentes dimensiones de la sostenibilidad, sin mencionar relaciones explícitas que desencadenen procesos de cambio dirigidos hacia ella (12), Pluye y colaboradores (13) y Atun y colaboradores (19) adoptan una perspectiva temporal evolutiva con estadios progresivos que caracterizan la maduración del proceso de sostenibilidad o de integración. Por último, Gruen y colaboradores (14) y Shigayeva y Coker (15) construyen marcos de análisis en los cuales se supone la existencia de relaciones entre los factores que influyen en la sostenibilidad sin considerar una predeterminación evolutiva del proceso desencadenado.

## DISCUSIÓN

En esta sección se interpreta la sostenibilidad del PAI a partir de los tres ejes analíticos que aparecen en la bibliografía analizada en el contexto de la Estrategia para el acceso universal a la salud y la cobertura universal de salud.

### La sostenibilidad y la integración como objeto de análisis

En relación con la sostenibilidad y la integración, dicha Estrategia permite incluir las dimensiones de integración del PAI como un factor facilitador de su sostenibilidad. Mientras que la sostenibilidad del PAI está asociada con una institucionalidad creciente de sus normas y estándares, y como parte de una agenda de fortalecimiento de los sistemas de salud (13, 19), su integración se analiza a partir de las dimensiones relacionadas con las distintas líneas de esta Estrategia.

La línea estratégica de ampliación del acceso a los servicios de salud, que promueve un modelo de atención integral e integrado y centrado en las personas y las comunidades, requiere un modelo de gestión del PAI con mecanismos de coordinación e integración con las estructuras organizativas con que se gestionan servicios de salud integrales, con población y territorio a cargo, especialmente en el primer nivel de atención, y entendiendo la inmunización como un componente central de la prevención primaria. En esta misma línea, el componente logístico de las vacunas es un elemento esencial del funcionamiento de los programas de inmunizaciones que puede fortalecerse si se logran sinergias y economías de escala con parte del resto de la logística del sistema de salud. Además, las normas de cobertura y los protocolos del programa de inmunizaciones son ingredientes complementarios del acervo de guías clínicas y normas de atención que los equipos de salud emplean como herramientas de trabajo y, por lo tanto, ofrecen oportunidades de mayor integración.

La línea estratégica de rectoría y gobernanza incluye la necesidad de fortalecer el papel de las autoridades de salud en los procesos de formulación de políticas y programas de salud. Tanto los procesos de formulación de las políticas de salud, como el diseño y la implementación de los mecanismos de monitorización deben abordarse de forma integral, y en ellos las intervenciones de promoción y prevención asociadas con el PAI han de estar integradas en los procesos de transformación de los sistemas y servicios de salud.

La línea estratégica asociada con la mejora y el aumento del financiamiento incluye la integración en el marco de innovaciones del modelo de gestión presupuestaria, para garantizar los recursos necesarios y alinear los incentivos que faciliten la mejor conformación y el mejor desempeño de las redes integradas de servicios de salud. De forma complementaria, se ha promovido la integración nacional y regional de las compras de vacunas, como ejemplifica el mecanismo del fondo rotatorio de la OPS, que, con el objetivo explícito de fortalecer los PAI de los Estados Miembros, ha permitido el acceso a vacunas a menores costos (20).

La línea estratégica que promueve la intersectorialidad tiene implicaciones importantes en este tema. El PAI ha sido una iniciativa emblemática en la generación de acciones intersectoriales al adoptar un abordaje territorial para avanzar con su efectividad (21). Las nuevas iniciativas intersectoriales que se avanzan desde los servicios de salud tienen mucho que recibir y ofrecer en instancias de integración con el PAI (22).

### Factores institucionales y políticos

Respecto a cómo influyen los factores institucionales y políticos en la sostenibilidad del PAI, la Estrategia, que afronta diversos desafíos, explicita como valores: el derecho a la salud, la solidaridad y la equidad de los procesos de transformación de los sistemas de salud. En este marco, los procesos políticos se consideran motores de las transformaciones institucionales necesarias para avanzar en este sentido.

La Estrategia también hace explícito un análisis crítico de las limitaciones institucionales de los sistemas de salud entendidas como segmentación o como fragmentación de la organización de los servicios de salud, junto con problemas manifiestos en el acceso y la calidad de los servicios. El PAI ha sido un ejemplo emblemático de extensión de la cobertura efectiva de vacunación en la Región para la población que vive en condiciones de pobreza y, por consiguiente, se ha convertido en una intervención crucial en la reducción de inequidades y en la extensión de un sistema de cobertura universal para toda la población. Actualmente, los desafíos institucionales y políticos se orientan hacia la necesidad de incluir los programas de inmunizaciones en una agenda de transformación de la organización de los servicios y del modelo de atención. Las diferentes dimensiones de integración del PAI, identificadas anteriormente, permiten reconocer oportunidades para abordar la fragmentación de la provisión de los servicios a través del fortalecimiento de redes integrales e integradas de servicios de salud.

Los factores políticos de la Estrategia se ven reflejados especialmente en el fortalecimiento de la función rectora de las autoridades de salud. En este marco es necesario reconocer que los desafíos de la sostenibilidad del PAI también deben interpretarse como parte de un proceso político en el cual se pone en juego la legitimación de un modelo de atención que responda a las necesidades de salud de la población y esté enmarcado en un sistema de protección social que garantice el derecho a la salud a través de la solidaridad y la equidad en el acceso a los servicios de salud. Para avanzar en esta dirección, la sostenibilidad política del PAI debe interpretarse desde el prisma de su integración en la implementación de la Estrategia a través de alianzas entre actores que permitan conformar un liderazgo colectivo suficiente.

### Los procesos de cambio

Aunque la bibliografía analizada ofrece dos perspectivas —una centrada en la caracterización del proceso de cambio y basada en la definición de etapas predefinidas (13, 19) y otra que resalta el componente dinámico (12, 14, 15)—, sus aportaciones no consiguen delinear la complejidad de los procesos de cambio de los sistemas de salud en la Región. La sostenibilidad del PAI es un proceso claramente concomitante con otros procesos, como el fortalecimiento de los servicios de salud y la transformación institucional de los sistemas de salud en su conjunto. Si no se incorpora esta perspectiva, se corre el riesgo de restringir este debate exclusivamente a un intento de sostener o procurar recursos financieros para el funcionamiento del PAI.

La Estrategia hace contribuciones importantes en esta materia, ya que en ella se supone que la sostenibilidad de los programas prioritarios debe afrontarse por medio del fortalecimiento y de la transformación de los sistemas de salud. Incluso aunque se supone que tales procesos deben formularse e implementarse considerando las características políticas e institucionales de cada país, también se subraya la importancia de monitorizar y evaluar los esfuerzos que se realicen en su despliegue a fin de identificar y caracterizar los avances, los desafíos y los nudos críticos de los procesos de transformación.

La identificación y la clasificación de las dimensiones de integración del PAI en el sistema de salud a partir de las líneas de la Estrategia pueden servir como unidad de análisis de la monitorización y evaluación de tales procesos de transformación. Una agenda de trabajo futura podría enfocarse a medir y caracterizar los avances mencionados que se realicen en cada una las dimensiones de integración del PAI en los sistemas de salud, en el contexto de la Estrategia y teniendo en cuenta la complejidad institucional y política en países con diferentes niveles de desarrollo de sus sistemas de salud.

Como conclusiones cabe decir, en primer lugar, que este artículo busca contribuir a un debate conceptual y práctico, todavía pendiente, sobre los desafíos del PAI en la Región de las Américas a través de la interpretación de las aportaciones de diferentes marcos analíticos publicados recientemente a partir del contexto de la Estrategia de acceso universal a la salud y cobertura universal de la salud. El abordaje utilizado permite sistematizar tres ejes analíticos.

En segundo lugar, aunque la integración y la sostenibilidad no se interpretan de forma homogénea en la bibliografía analizada, se ponderan las virtudes de un enfoque que conciba la integración como un factor facilitador del proceso de sostenibilidad del PAI. Con este abordaje se reconocen opciones de políticas de integración del PAI asociadas con las diferentes líneas de la Estrategia de salud universal. En este marco, se enfatiza también la necesidad de reconocer la dinámica del proceso de integración y la sostenibilidad del PAI asociado con el fortalecimiento y transformación del sistema de salud en su conjunto.

En tercer lugar, la integración del PAI en el sistema de salud y la construcción de la sostenibilidad de sus avances para hacer frente a los desafíos actuales y pendientes son condiciones que no se producen en el vacío. Por el contrario, la efectividad de tales estrategias depende de la presencia, el fortalecimiento y la transformación de estructuras organizativas e institucionales que faciliten la participación de los actores con capacidad técnica y política para garantizar sus procesos de implementación. De esta forma, se ponderan los procesos políticos que legitiman una agenda de inclusión del PAI en la Estrategia de salud universal y, por lo tanto, como parte del fortalecimiento de los sistemas de salud.

Por último, la sostenibilidad se entiende como un proceso en construcción, de transición y contingente respecto a las características de cada contexto particular. Este factor tiene una relación directa con la Estrategia de salud universal por su carácter de proceso y situacional y, por ello, es necesario reconocer en cada escenario nacional y subnacional cuáles son los desafíos del PAI para integrarse en una agenda de ampliación de las condiciones de acceso y cobertura universales.

### Financiación.

Este estudio no ha recibido financiación.

### Declaración.

 Las opiniones expresadas en este manuscrito son responsabilidad del autor y no reflejan necesariamente los criterios ni la política de la RPSP/PAJPH y/o de la OPS.
